# Assessment of Salivary Neutrophil Gelatinase-Associated Lipocalin (NGAL) Levels Among Subjects With Oral Squamous Cell Carcinoma, Periodontitis, Smokeless Tobacco Users, Smokers, and Healthy Individuals: A Cross-Sectional Study

**DOI:** 10.7759/cureus.110999

**Published:** 2026-06-16

**Authors:** Sayali S Dongare, Sameer A Zope, Girish Suragimath, Siddhartha Varma, Apurva V Kale

**Affiliations:** 1 Periodontology, School of Dental Sciences, Krishna Vishwa Vidyapeeth (Deemed to be University), Karad, IND

**Keywords:** neutrophil gelatinase-associated lipocalin, oral squamous cell carcinoma, periodontitis, saliva, smoked tobacco, smokeless tobacco

## Abstract

Background: Neutrophil gelatinase-associated lipocalin (NGAL) is an inflammatory biomarker implicated in periodontal tissue destruction and tumor progression. Tobacco use, both smoked and smokeless, is a key risk factor for periodontitis and oral squamous cell carcinoma (OSCC) and may influence NGAL expression. However, comparative evaluation of salivary NGAL levels across periodontal disease, tobacco users, and OSCC remains limited.

Aim: To compare salivary NGAL levels among patients with OSCC, periodontitis, smoked tobacco users, smokeless tobacco (SLT) users, and healthy controls, and to assess the correlation of NGAL levels with periodontal clinical parameters.

Materials and methods: This cross-sectional study included 125 participants divided into five groups (n = 25 each): OSCC, periodontitis, smokers, SLT users, and healthy controls. Periodontal parameters such as Oral Hygiene Index (OHI), Russell’s Periodontal Index, probing pocket depth (PPD), and clinical attachment level (CAL) were recorded. Unstimulated whole saliva samples were collected and analyzed for NGAL levels using enzyme-linked immunosorbent assay (ELISA). Statistical analysis was performed using analysis of variance (ANOVA) with Tukey’s post-hoc test and Pearson’s correlation analysis (p < 0.05).

Results: Salivary NGAL levels showed a statistically significant difference among all groups (p < 0.001), with the highest levels observed in SLT users, followed by patients with OSCC, patients with periodontitis, smoked tobacco users, and healthy controls. Periodontal parameters were significantly worse in patients with periodontitis and SLT users, with no significant difference between these two groups. Salivary NGAL exhibited strong positive correlations with OHI, Russell’s Index, PPD, and CAL (p < 0.001).

Conclusion: Elevated salivary NGAL levels are significantly associated with periodontal disease severity, both smoked and SLT use, and OSCC. NGAL shows promise as a potential non-invasive biomarker for assessing inflammatory burden and disease risk in periodontal and tobacco-related oral conditions.

## Introduction

Oral health is a critical component of overall well-being, yet a significant proportion of the global population suffers from a wide range of oral diseases [1]. Among these conditions, periodontitis and oral squamous cell carcinoma (OSCC) are among the most prevalent and clinically significant disorders. Although these diseases represent opposite ends of the pathological spectrum - chronic inflammation and malignancy - they share several underlying biological mechanisms, including inflammation, immune dysregulation, and extracellular matrix degradation. These processes are particularly pronounced in individuals with prolonged tobacco exposure, a major risk factor for both conditions.

Periodontitis is a chronic, multifactorial inflammatory disease affecting the supporting structures of the teeth, including the gingiva, periodontal ligament, and alveolar bone [2]. The disease is primarily initiated by microbial dysbiosis, with key pathogens, such as *Porphyromonas gingivalis*, *Tannerella forsythia*, and *Treponema denticola*, triggering a host immune response [3,4]. While this response initially serves a protective function, persistent stimulation can lead to immune dysregulation and progressive tissue destruction. Pro-inflammatory cytokines, including interleukin (IL)-1β, tumor necrosis factor (TNF)-α, and IL-6, together with matrix metalloproteinases (MMPs) and prostaglandins, promote collagen degradation and alveolar bone resorption, resulting in progressive periodontal breakdown [5].

The global burden of periodontitis remains substantial. According to the Global Burden of Disease Study, severe periodontitis affects more than 796 million individuals worldwide and ranks as the eleventh most prevalent disease [6]. The condition is particularly common in developing countries, including those in South Asia, where poor oral hygiene practices, limited access to dental care, and high tobacco consumption contribute to disease prevalence. In India, periodontitis is estimated to affect 40%-70% of adults, with higher prevalence reported among older individuals, males, and rural populations. To improve diagnosis and management, the 2017 World Workshop classification introduced a comprehensive staging and grading framework that incorporates major risk factors, such as smoking and diabetes [7].

Tobacco use remains the most important modifiable risk factor for both periodontitis and OSCC. Smoking adversely affects immune cell function, reduces vascular perfusion, alters the subgingival microbiota, and inhibits fibroblast activity. Consequently, smokers often exhibit greater periodontal destruction, with disease severity increasing in proportion to the duration and intensity of tobacco exposure. Smoked tobacco products, including cigarettes, bidis, and cigars, introduce carcinogenic compounds, such as polycyclic aromatic hydrocarbons (PAHs), nitrosamines, and reactive oxygen species (ROS), into the oral cavity. These agents damage oral tissues, impair host defenses, and delay tissue repair [8,9]. Furthermore, smoking creates a hypoxic environment within the gingival tissues, favoring anaerobic bacterial growth and hindering healing.

Smokeless tobacco (SLT), which is widely consumed throughout South Asia, also contributes significantly to periodontal destruction. Its use is associated with localized gingival recession, alveolar bone loss, and tissue damage at habitual placement sites. Beyond its effects on periodontal health, tobacco is one of the major etiological factors for OSCC, a malignant epithelial neoplasm characterized by local invasion, metastasis, and high recurrence rates. The pathogenesis of OSCC involves a complex interplay of genetic and epigenetic alterations, chronic inflammation, and environmental carcinogens, all of which are exacerbated by tobacco exposure [10,11].

Several tobacco-derived carcinogens, including benzo[a]pyrene and N-nitrosonornicotine, induce DNA damage, disrupt cell-cycle regulation, and impair immune surveillance mechanisms [12,13]. These changes facilitate tumor initiation and progression while promoting immune evasion. In addition, inflammatory mediators, such as ILs, TNF-α, and prostaglandins, contribute to extracellular matrix degradation and epithelial-mesenchymal transition (EMT), both of which are critical processes in tumor invasion and metastasis.

A molecule of growing interest in both periodontitis and OSCC is neutrophil gelatinase-associated lipocalin (NGAL), also known as lipocalin-2 (LCN2). Initially identified in neutrophils, NGAL is also expressed by epithelial cells, hepatocytes, and renal tubular cells in response to inflammatory stimuli. It serves a dual biological function by limiting microbial iron acquisition as part of innate immunity while also influencing cell proliferation, apoptosis, and matrix remodeling.

In periodontitis, NGAL is released from activated neutrophils within inflamed periodontal tissues, and elevated concentrations have been detected in both saliva and gingival crevicular fluid. In OSCC, altered NGAL expression has been associated with tumor burden, inflammatory activity, and disease progression. Furthermore, by stabilizing MMP-9, NGAL may enhance extracellular matrix degradation and EMT, processes implicated in both chronic inflammatory tissue destruction and tumor invasion. These observations suggest that NGAL may represent a shared biological marker of tissue inflammation and remodeling across a spectrum of oral diseases rather than a disease-specific indicator.

The present cross-sectional study was designed to evaluate salivary NGAL levels across five distinct cohorts: patients with OSCC, patients with chronic periodontitis, habitual smokers, SLT users, and healthy controls. These groups were selected to represent varying degrees and sources of oral inflammatory burden, ranging from clinically healthy individuals to subjects with tobacco exposure, chronic periodontal inflammation, and malignant disease. By comparing NGAL expression across this spectrum, the study aims to explore whether salivary NGAL primarily reflects underlying inflammatory and tissue-destructive processes common to these conditions.

In addition, the study seeks to examine the relationship between salivary NGAL concentrations and established periodontal clinical parameters, thereby evaluating the extent to which NGAL levels correspond with the severity of periodontal inflammation and tissue destruction. A comparison of smokers and SLT users may further provide insight into the influence of different forms of tobacco exposure on oral inflammatory status. Collectively, these analyses may improve understanding of the biological significance of salivary NGAL in oral disease and clarify its potential utility as a marker of local inflammatory burden within the oral cavity.

## Materials and methods

**Figure 1:**
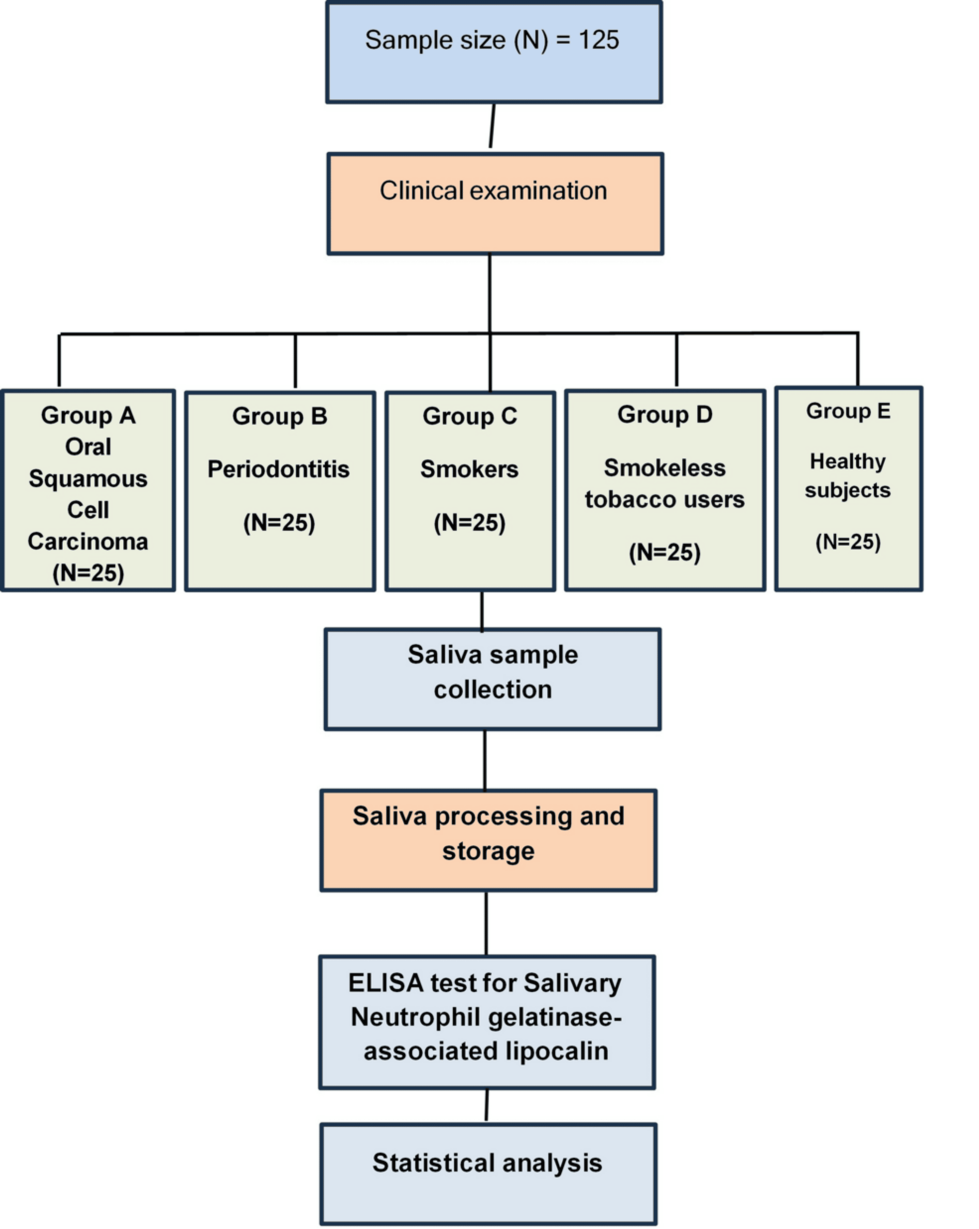
Summary of the study protocol ELISA: enzyme-linked immunosorbent assay

The present cross-sectional study was conducted in the Department of Periodontology, School of Dental Sciences (KVV), Krishna Vishwa Vidyapeeth, Karad (Figure 1). Ethical clearance was obtained from the Institutional Ethics Committee (IEC approval number: KIMSDU/IEC/06/2023).

Sample size selection

A total sample size of 125 was selected using a purposive sampling technique. The required sample size was determined through power analysis, yielding a minimum of 125 participants (25 per group across five groups), which provided 95% statistical power to detect significant differences at a 5% level of significance. 

The sample size was calculated using the following formula:

$n = \frac{(Z_{\alpha} + Z_{\beta})^{2}\left(S_{1}^{2} + S_{2}^{2}\right)}{(m_{2} - m_{1})^{2}}$

Here, n is the required sample size per group; $Z_{\alpha}$ is the standard normal deviate corresponding to the selected significance level $\alpha)$; $Z_{\beta}$ is the standard normal deviate corresponding to the desired statistical power; $S_1^2$ is the variance of the outcome in Group 1; $S_2^2$ is the variance of the outcome in Group 2; $S1, S2$ are standard deviations of the outcome in Group 1 and Group 2, respectively; $m_1$ is the expected mean value of the outcome in Group 1; $m_2$ is the expected mean value of the outcome in Group 2; and $m_2 - m_1$ is the minimum difference in means (effect size) that the study aims to detect.

Inclusion criteria

All participants provided informed consent before enrollment in the study. Participants aged 20-60 years, both male and female, were included and divided into five groups:

1) Group A: patients with untreated OSCC confirmed clinically and histopathologically

2) Group B: subjects with generalized periodontitis

3) Group C: current smokers consuming ≥five cigarettes/day

4) Group D: users of SLT (gutkha, pan masala, and khaini) consuming ≥ one packet/day for ≥ six months

5) Group E: systemically healthy individuals with no tobacco use or adverse habits

Exclusion criteria

Patients with medical conditions requiring antibiotic prophylaxis; those with systemic or metabolic disorders, such as diabetes, immunological diseases, alcoholism, or illicit drug use; individuals taking anti-inflammatory, anticonvulsant, immunosuppressant, vitamin supplements, or calcium channel blockers; patients who had received periodontal therapy or medications affecting periodontal tissues in the last six months; and those who did not provide informed consent were excluded from the study.

Clinical examination

Participants underwent a detailed oral examination to assess mucosal lesions, masses, and lymph node status, with clinical staging and histopathological confirmation for OSCC cases. Periodontal parameters, including Oral Hygiene Index, Gingival Index, Russell’s Periodontal Index, probing depth, attachment level, and bleeding on probing, were recorded by a calibrated examiner using the UNC-15 periodontal probe. Radiographs were used to assess bone loss. Tobacco use history and related oral changes were also documented.

Salivary sample collection

Unstimulated whole saliva (2 mL) was collected from each participant between 9:00 AM and 12:00 PM, following a modified protocol described by Navazesh to minimize the effects of circadian fluctuations on salivary flow rate and biomarker levels [8]. Participants were instructed to abstain from eating, drinking, and oral hygiene procedures for at least one hour before sample collection. To minimize the risk of blood contamination, all clinical periodontal measurements were performed at least one hour before saliva collection. Subjects were first asked to swallow residual saliva, after which unstimulated saliva was allowed to passively pool and drain over the lower lip for five minutes into a sterile collection tube. Samples were then appropriately stored for subsequent analysis. 

Sample storage

Immediately after the collection of samples, they were centrifuged for 15 minutes at 1,000 rpm at 2-8°C. Following centrifugation, all particulate matter was removed, and the clarified supernatant was aliquoted. The saliva samples were immediately stored at -80°C until further analysis. All samples were analyzed within two months of collection.

Biomarker analysis

The concentration of salivary NGAL was determined using enzyme-linked immunosorbent assay (ELISA) kits (EasyStep Human NGAL ELISA Kit; ELK Biotechnology, USA). The analysis was conducted at the Microbiology Laboratory, Krishna Vishwa Vidyapeeth Deemed to be University, Karad.

Statistical analysis

The biomarker data were subjected to statistical analysis using the Statistical Package for the Social Sciences (SPSS), version 21.0 (released 2012; IBM Corp., Armonk, NY, USA). Intergroup comparisons of the measured parameters were conducted using one-way analysis of variance (ANOVA), followed by Tukey’s post-hoc test for multiple pairwise comparisons. Associations between variables were evaluated using the Pearson correlation coefficient. Statistical significance was defined at p < 0.05.

## Results

A total of 125 subjects were included using the purposive sampling method and divided into five groups: the OSCC group (Group A, n = 25), the periodontitis group (Group B, n = 25), the smoker group (Group C, n = 25), the SLT user group (Group D, n = 25), and the healthy control group (Group E, n = 25). Group B had the highest mean age (48.04 years), followed closely by Group A (47.64 years). Group D had a mean age of 44.24 years, Group C had 37.08 years, and Group E had the lowest mean age at 33.2 years (Table 1). Among males, there were 19 participants in Group A, 17 in Group B, 25 in Group C, 20 in Group D, and 14 in Group E. Among females, there were 6 participants in Group A, 8 in Group B, none in Group C, 5 in Group D, and 11 in Group E.

**Table 1:** Comparison of mean age and gender distribution among the study groups **Statistically highly significant (p<0.001) SD: standard deviation; SE: standard error

Groups	Mean age (years)	SD	SE	Minimum	Maximum	Male N (%)	Female N (%)	F-value	p-value
Group A	47.64	7.49	1.50	40.15	55.13	19 (76)	6 (24)	13.7	0.000**
Group B	48.04	7.45	1.49	40.59	55.49	17 (68)	8 (32)		
Group C	37.08	10.65	2.13	26.43	47.73	25 (100)	0 (0)		
Group D	44.24	10.52	2.10	33.72	54.76	20 (80)	5 (20)		
Group E	33.20	8.72	1.74	24.48	41.92	14 (56)	11 (44)		

Table 2 describes the comparative analysis of periodontal parameters across the five study groups. All periodontal clinical parameters differed significantly among the study groups (p < 0.001). The highest mean OHI, Russell's Index, PPD, and CAL values were observed in the periodontitis (Group B) and SLT user groups (Group D). Healthy controls (Group E) exhibited the lowest values for all parameters, while smokers (Group C) showed intermediate values. Overall intergroup differences were statistically significant for OHI and Russell's Index (p = 0.0004 each), as well as PPD and CAL (p = 0.0001 each).

**Table 2:** Comparison of periodontal clinical parameters among study groups Data are presented as mean ± standard deviation (SD). One-way ANOVA was used for intergroup comparison. *Statistically significant difference (p < 0.05) OHI: Oral Hygiene Index; PPD: probing pocket depth; CAL: clinical attachment level; OSCC: oral squamous cell carcinoma; SLT: smokeless tobacco; ANOVA; analysis of variance

Parameter	Group A (OSCC)	Group B (periodontitis)	Group C (smokers)	Group D (SLT users)	Group E (healthy controls)	p-value*
OHI	3.50 ± 0.35	3.90 ± 0.38	2.10 ± 0.30	3.85 ± 0.36	0.65 ± 0.20	0.0004*
Russell's Index	3.40 ± 0.41	4.00 ± 0.43	2.00 ± 0.38	3.95 ± 0.40	0.30 ± 0.15	0.0004*
PPD (mm)	6.20 ± 0.55	6.90 ± 0.65	5.00 ± 0.50	6.85 ± 0.60	0.00 ± 0.00	0.0001*
CAL (mm)	3.90 ± 0.65	4.80 ± 0.75	2.80 ± 0.60	4.75 ± 0.70	0.00 ± 0.00	0.0001*

Table 3 summarizes the Tukey post-hoc pairwise comparisons for all periodontal parameters. Significant differences were observed between most group pairs for OHI, Russell's Index, PPD, and CAL (p < 0.001). However, no statistically significant differences were detected between the periodontitis group (Group B) and the SLT user group (Group D) for any periodontal parameter (OHI: p = 0.880; Russell's Index: p = 0.950; PPD: p = 0.970; CAL: p = 0.990), indicating comparable periodontal tissue destruction in these two groups. Healthy controls (Group E) differed significantly from all disease and tobacco-exposed groups (p < 0.001). Similarly, smokers (Group C) exhibited significantly lower periodontal parameter values than Groups A, B, and D but significantly higher values than healthy controls. These findings suggest that periodontitis and SLT use were associated with the greatest periodontal impairment among the study groups.

**Table 3:** Pairwise comparison of periodontal parameters among study groups using Tukey’s post-hoc test Tukey's post-hoc test following one-way ANOVA. Statistical significance was set at p < 0.05. S: statistically significant difference; NS: not statistically significant difference; A: OSCC; B: periodontitis; C: smokers; D: smokeless tobacco users; E: healthy controls; PPD: probing pocket depth; CAL: clinical attachment level; OHI: Oral Hygiene Index

Comparison groups	OHI	Russell's Index	PPD (mm)	CAL (mm)
A vs. B	S (p<0.001)	S (p<0.001)	S (p<0.001)	S (p<0.001)
A vs. C	S (p<0.001)	S (p<0.001)	S (p<0.001)	S (p<0.001)
A vs. D	S (p<0.001)	S (p<0.001)	S (p<0.001)	S (p<0.001)
A vs. E	S (p<0.001)	S (p<0.001)	S (p<0.001)	S (p<0.001)
B vs. C	S (p<0.001)	S (p<0.001)	S (p<0.001)	S (p<0.001)
B vs. D	NS (p=0.880)	NS (p=0.950)	NS (p=0.970)	NS (p=0.990)
B vs. E	S (p<0.001)	S (p<0.001)	S (p<0.001)	S (p<0.001)
C vs. D	S (p<0.001)	S (p<0.001)	S (p<0.001)	S (p<0.001)
C vs. E	S (p<0.001)	S (p<0.001)	S (p<0.001)	S (p<0.001)
D vs. E	S (p<0.001)	S (p<0.001)	S (p<0.001)	S (p<0.001)

**Figure 2:**
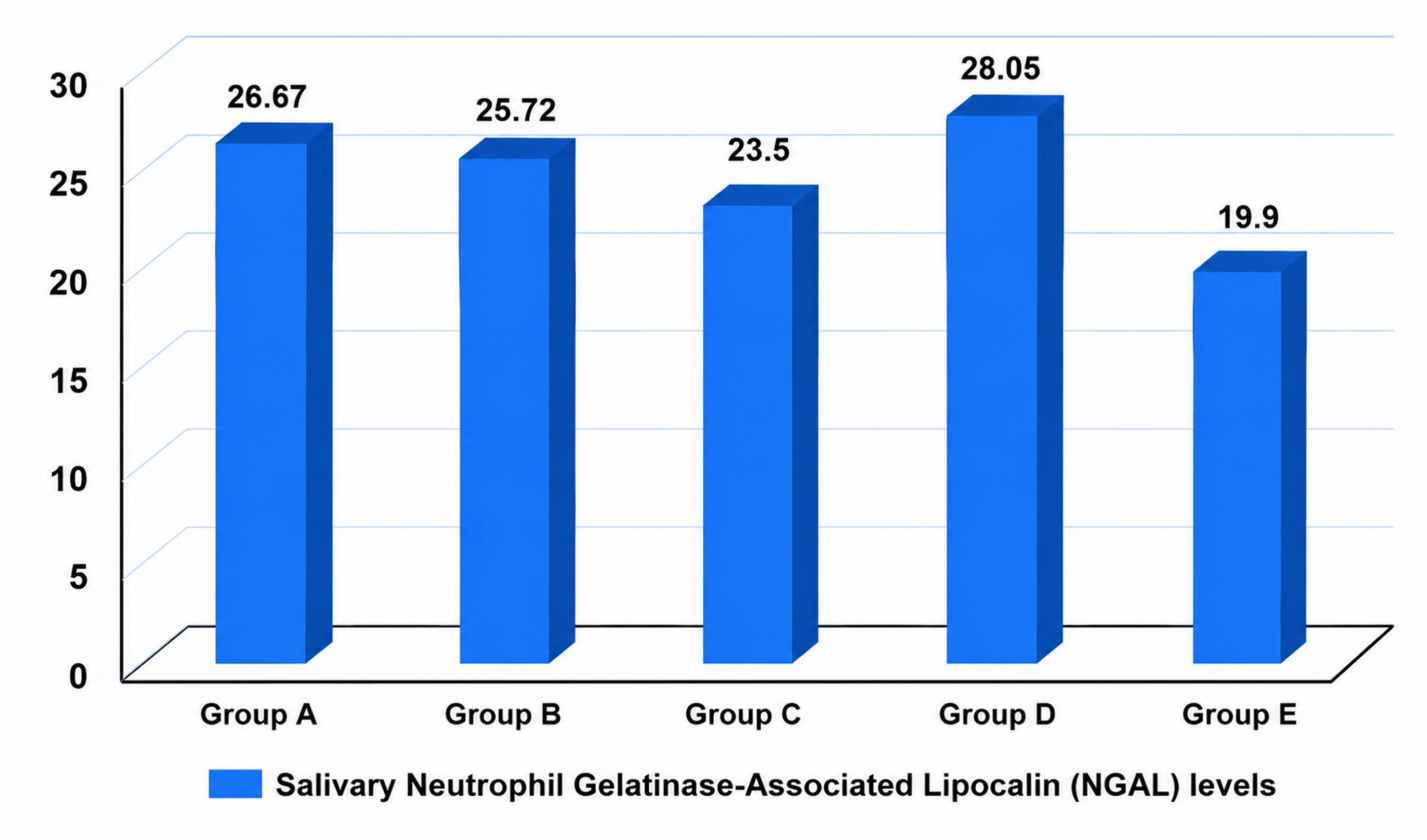
Mean salivary NGAL levels among different groups The X-axis represents the study groups. The Y-axis represents the mean salivary neutrophil gelatinase-associated lipocalin (NGAL) levels in ng/mL.

Figure 2 illustrates the distribution of salivary NGAL concentrations across the five study groups. Group A showed a mean of 26.67 ng/mL, Group B 25.72 ng/mL, Group C 23.5 ng/mL, Group D had the highest at 28.05 ng/mL, and Group E had the lowest at 19.9 ng/mL. The observed differences in salivary NGAL concentrations among the groups were statistically highly significant (F = 277.36, p < 0.001), reflecting a clear association between increased NGAL levels and the presence or severity of oral disease and tobacco exposure.

Table 4 presents the Tukey post-hoc comparisons of salivary NGAL levels among the study groups. Significant differences were observed between all pairwise comparisons (p < 0.001). The largest difference was observed between SLT users (Group D) and healthy controls (Group E) (mean difference: 8.15 ng/mL), whereas the smallest difference was observed between the OSCC (Group A) and periodontitis (Group B) groups (mean difference: 0.95 ng/mL). Overall, salivary NGAL levels were significantly elevated in tobacco users, patients with periodontitis, and patients with OSCC compared with healthy controls.

**Table 4:** Pairwise comparison of mean salivary NGAL levels among study groups using Tukey’s post-hoc test Statistical significance was set at p < 0.05. A: OSCC; B: periodontitis; C: smokers; D: smokeless tobacco users; E: healthy controls; NGAL: neutrophil gelatinase-associated lipocalin

Comparison groups	Mean difference (ng/mL)	p-value
A vs. B	0.95	<0.001
A vs. C	3.17	<0.0001
A vs. D	1.38	<0.0001
A vs. E	6.77	<0.0001
B vs. C	2.22	<0.0001
B vs. D	2.33	<0.0001
B vs. E	5.82	<0.0001
C vs. D	4.55	<0.0001
C vs. E	3.6	<0.0001
D vs. E	8.15	<0.0001

Table 5 revealed strong and statistically significant positive correlations among all clinical periodontal parameters, including the OHI, Russell’s Index, PPD, and CAL. Notably, the correlation between PPD and CAL was the highest (r = 0.902, p = 0.0001), followed by strong correlations between OHI and Russell’s Index (r = 0.876, p = 0.0001), OHI and PPD (r = 0.852, p = 0.0001), and Russell’s Index and CAL (r = 0.861, p = 0.0001). These findings indicate that these parameters are closely related and tend to increase simultaneously with the progression of periodontal disease. Furthermore, salivary NGAL levels demonstrated moderate to strong positive correlations with all clinical indicators of periodontal status, including OHI (r = 0.701, p = 0.001), Russell’s Index (r = 0.687, p = 0.0001), PPD (r = 0.722, p = 0.0001), and CAL (r = 0.734, p = 0.0001).

**Table 5:** Pearson’s correlation between various variables PPD: probing pocket depth; CAL: clinical attachment level; OHI: Oral Hygiene Index; NGAL: neutrophil gelatinase-associated lipocalin

Variables	OHI	Russell’s Index	PPD	CAL	NGAL
OHI	1	0.876**	0.852**	0.833**	0.701**
p-value	-	0.0001	0.0001	0.0001	0.0001
Russell’s Index	0.876**	1	0.891**	0.861**	0.687**
p-value	0.0001	-	0.0001	0.0001	0.0001
PPD	0.852**	0.891**	1	0.902**	0.722**
p-value	0.0001	0.0001	-	0.0001	0.0001
CAL	0.833**	0.861**	0.902**	1	0.734**
p-value	0.0001	0.0001	0.0001	-	0.0001
NGAL	0.701**	0.687**	0.722**	0.734**	1
p-value	0.0001	0.0001	0.0001	0.0001	-

## Discussion

Periodontitis is a chronic inflammatory condition characterized by progressive destruction of the tooth-supporting apparatus, including the periodontal ligament and alveolar bone [14]. The apical migration of the junctional epithelium and subsequent formation of periodontal pockets are hallmark features of the disease. Bacterial pathogens, especially gram-negative anaerobes such as *Porphyromonas gingivalis*, trigger the host immune response by releasing virulence factors, including lipopolysaccharides (LPS), fimbriae, and capsular polysaccharides [15]. These components activate pattern recognition receptors such as Toll-like receptors (TLRs), which in turn initiate intracellular signaling cascades, leading to the release of pro-inflammatory cytokines, such as IL-1β, TNF-α, and IL-6. These molecules play critical roles in leukocyte recruitment, vascular changes, and upregulation of MMPs, promoting tissue destruction [16]. In parallel, prostaglandin E2 (PGE2) and leukotriene B4 (LTB4) further exacerbate inflammation and bone resorption, driving the chronicity of the disease.

NGAL, a member of the lipocalin protein family, has emerged as a multifaceted biomarker with roles in antimicrobial defense, iron metabolism, and tissue remodeling. Structurally characterized by an eight-stranded β-barrel, NGAL binds low-molecular-weight ligands, including bacterial siderophores, thereby limiting bacterial proliferation through iron sequestration. NGAL exists in monomeric (30 kDa), dimeric (46 kDa), and MMP-9-complexed (130 kDa) forms, and is expressed in various cell types, such as neutrophils, renal tubular cells, hepatocytes, and epithelial cells [17]. Functionally, NGAL interacts with eukaryotic ligands and plays a role in oxidative stress modulation and epithelial cell proliferation. Its oncogenic properties stem largely from its ability to stabilize MMP-9, enhancing extracellular matrix degradation and facilitating tumor invasion and metastasis. NGAL is detectable in saliva, serum, urine, and gingival crevicular fluid, making it a promising non-invasive biomarker in periodontal and malignant diseases of the oral cavity.

Tobacco consumption, both smoked and smokeless, remains the most significant environmental risk factor for periodontal disease and oral cancer. Nicotine adversely affects immune response, impairs neutrophil function, reduces phagocytosis, and alters cytokine profiles, including suppression of protective ILs and upregulation of tissue-destructive mediators such as TNF-α and PGE2. SLT, widely used in South Asia, contains high concentrations of carcinogens and alkaloids that lead to local gingival recession, periodontal attachment loss, and tissue inflammation [18]. Cigarette smoking similarly compromises host immunity by reducing T-cell proliferation, antibody production, and monocyte function while paradoxically increasing destructive inflammatory cytokines. These immunoinflammatory changes caused by tobacco products contribute significantly to periodontal breakdown and predispose the oral mucosa to malignant transformation.

OSCC, the most common oral malignancy, develops through a multistep process of genetic and epigenetic alterations induced by chronic exposure to carcinogens, particularly tobacco-related compounds. Substances such as tobacco-specific nitrosamines (N-nitrosonornicotine (NNN) and nitrosamine ketone (NNK)), polycyclic aromatic hydrocarbons, formaldehyde, and acetaldehyde are directly implicated in DNA damage, gene mutation, and oxidative stress [19]. OSCC is associated with dysregulation of key signaling pathways, including EGFR, PI3K/AKT, and nuclear factor (NF)-κB, along with the inactivation of tumor suppressor genes, such as* TP53* and *CDKN2A*. These molecular alterations collectively contribute to epithelial dysplasia, enhanced cellular proliferation, and metastatic potential. The role of NGAL in cancer is underscored by its interaction with MMP-9, which enhances tumor cell invasion and resistance to apoptosis. NGAL expression has been variably reported in OSCC tissues, suggesting both tumor-promoting and protective roles depending on disease stage and microenvironmental context.

The present study evaluated salivary NGAL levels among five groups: patients with OSCC, patients with periodontitis, cigarette smokers, SLT users, and healthy controls. The highest NGAL levels were found in Group D (SLT users), followed by Group A (OSCC), Group B (periodontitis), Group C (smokers), and Group E (healthy controls). This pattern suggests a progressive increase in inflammatory burden across the study groups, with particularly elevated levels among SLT users.

Group B demonstrated the highest mean age, consistent with the finding of Clark et al., who reported age-related increases in periodontal disease severity. Male predominance in the OSCC group was also observed, corroborating the findings of Johnson et al., who noted significantly higher OSCC incidence among males, especially in Southeast Asia [20,21].

OHI scores were the highest in Group B and Group D, reflecting poorer oral hygiene among patients with periodontitis and SLT users. These findings are in agreement with those of Ahad et al. and Yaragani et al., who reported a strong association between poor oral hygiene, tobacco use, and periodontal deterioration [22,23].

Similarly, periodontal clinical parameters, including PPD, CAL, and Russell’s Index, were the highest in Group D and Group B, indicating more advanced periodontal destruction. These observations align with previous reports by Madi et al. and Mehta et al., which emphasized the detrimental effects of SLT on periodontal tissues [24,25]. Although Tan et al. found no significant association between SLT use and periodontitis, the majority of available evidence, including the present findings, supports a strong positive relationship [26].

NGAL levels demonstrated a strong positive correlation with all clinical periodontal parameters, reinforcing its potential role as an inflammatory biomarker associated with periodontal disease severity. These findings are consistent with those of Mutar and Mahmood and Mohamad et al., who similarly reported elevated NGAL concentrations in patients with active periodontal disease [27,28].

Salivary NGAL concentrations were also elevated in patients with OSCC, supporting a potential role for NGAL in tumor-associated biological processes. While Yuan reported increased serum NGAL levels in OSCC and Monisha et al. observed downregulated tissue NGAL expression in OSCC, studies investigating salivary NGAL remain limited [29,30]. The present study contributes additional evidence regarding salivary NGAL expression in OSCC and highlights its possible relevance within the oral tumor microenvironment.

Notably, the highest NGAL concentrations were observed in SLT users, exceeding those detected in the OSCC group. This finding suggests that NGAL may reflect a generalized inflammatory response rather than a malignancy-specific signal. The markedly elevated levels among SLT users may be attributable to chronic mucosal irritation, epithelial injury, and persistent inflammatory activation induced by tobacco constituents. Furthermore, no previous studies have specifically evaluated salivary NGAL levels in SLT users, making this observation a novel contribution of the present investigation.

Taken together, these findings indicate that NGAL is closely associated with oral inflammatory burden and tissue destruction across diverse clinical conditions. The observed elevations in periodontitis, SLT use, and OSCC suggest that NGAL may function as a broad marker of inflammatory activity rather than a specific diagnostic biomarker for OSCC. Nevertheless, its consistent association with clinical disease parameters highlights its potential utility in assessing disease severity and monitoring inflammatory changes within the oral cavity. The present study has several limitations that should be considered when interpreting the findings. The relatively small sample size and cross-sectional design limit the generalizability of the results and preclude causal inferences. Additionally, demographic differences among the study groups, including the exclusively male smoker cohort and the younger age of healthy controls compared with the disease groups, may have influenced salivary NGAL levels and acted as potential confounding factors. Furthermore, the OSCC cohort was analyzed as a single group without stratification based on the tumor, node, metastasis (TNM) stage, histopathological grade, or primary tumor site, preventing assessment of the relationship between NGAL levels and tumor burden, disease severity, or clinicopathological characteristics. The absence of longitudinal follow-up and tissue-level NGAL evaluation further limits understanding of biomarker dynamics and local tissue expression.

Despite these limitations, the study provides valuable preliminary evidence regarding the association of salivary NGAL with periodontal disease, tobacco exposure, and OSCC. Future studies incorporating larger and demographically balanced cohorts, detailed clinicopathological characterization of OSCC, longitudinal follow-up, and tissue-based analyses are warranted to further clarify the role of NGAL as a surrogate marker of oral inflammatory burden and disease progression.

## Conclusions

This study demonstrated that salivary NGAL levels are elevated in periodontal disease, tobacco users, and patients with OSCC, with the highest concentrations observed in SLT users. These findings suggest that NGAL reflects the overall burden of oral inflammation and tissue destruction rather than being specific to malignancy. Although chronic periodontal inflammation and tobacco exposure may contribute to oral carcinogenesis, the present results do not support the use of salivary NGAL as a specific screening biomarker for OSCC. Instead, NGAL may serve as a broad surrogate marker of oral inflammatory status, warranting further investigation in larger and clinically stratified populations.
